# When Antibiotics Appear to Fail

**DOI:** 10.1155/S1064744997000045

**Published:** 1997

**Authors:** Gilles R. G. Monif

**Affiliations:** Department of Obstetrics and Gynecology Creighton University School of Medicine 601 North 30th Street Suite 4700 Omaha NE 68131 USA


Infectious Diseases in Obstetrics and Gynecology 5:8–9 (1997)
© 1997 Wiley-Liss, Inc.

### When Antibiotics Appear to Fail

**Gilles R.G. Monif, M.D.**
*Department of Obestetrics and Gynecology, Creighton University School of Medicine, Omaha, NE*

The patient was a 17-year-old unmarried, primigravida who delivered via vacuum-assisted vaginal extraction two days previously. In the course of the delivery, a significant midline and extensive right lateral wall vaginal tear occurred. The patient was discharged on the first postpartum day; however, she represented herself to the clinic 24 h later with shaking chills, a temperature of 103.3°F, and bilaterally costovertebral angle (CVA) tenderness. The patient denied any dysuria, vomiting, episiotomy pain, abdominal pain, foul smelling discharge, or breast tenderness.

Physical examination revealed a temperature of 103.3°F, pulse 110, respirations 16, blood pressure 110/70. Pertinent physical findings were limited to the abdominal and pelvic examinations. The abdomen was soft and nontender. No masses were present. Bowel sounds were present. The fundus was firm, 3 cm below the umbilicus, and nontender. The patient had marked CVA tenderness to minimal palpation. The remainder of the physical examination was unremarkable. Laboratory evaluation reveals a white count of 18,700, hematocrit 20.6, platelets 215,000. Urinalysis revealed 50–90 WBCs with positive nitrite and WBC esterase as well as many bacteria were seen.

### HOSPITAL COURSE

The patient was admitted with a diagnosis of postpartum pyelonephritis. Urine and blood cultures were obtained and the patient was started on intravenous ampicillin and gentamicin. While in the hospital, the patient continued to have spiking chills and fevers up to 102.5°F for the first 24 hours. Because of these temperature spikes on the night of first hospital admission, clindamycin was added to the intravenous antibiotic regimen. On the second day the patient continued to have fevers to 101.2°F. Urine culture at this point grew out $10^5$ cfu of *Escherichia coli* which was uniformly sensitive to ampicillin and gentamycin. The reexamination revealed no changes in the demonstration of the costovertebral angle tenderness, a minimal amount of swelling on the left vaginal wall; the uterus was nontender, and the fundus was firm. Despite three full days of intravenous antibiotic therapy, the patient continued to spike temperatures of 103.4°F. Ultrasound of the abdomen and pelvis revealed mild fullness in the right collective system which was deemed normal physiological postpartum state. There was a small amount of fluid and debris in the lower uterine endometrial cavity which was also consistent with postpartum findings. Triple antibiotic coverage was continued.

On the fourth hospital day the patient continued to spike a temperature of 101°F. At this point an Infectious Disease consultation was obtained from the OB/GYN Department. Pelvic and speculum examination revealed no evidence of hematoma or infection. Abdominal wall sutures were intact and well approximated. The uterus was nontender without cervical motion tenderness. No CVA tenderness was demonstrated. A repeat white blood cell count was 8,400, hematocrit 20, platelets 374,000.

The Infectious Disease consultant ordered the following: (1) Cultures of blood and urine for test of cure, and (2) Discontinuation of all antibiotics.

### OUTCOME

The patient became afebrile in six h and was discharged in 24 h.

### DIAGNOSIS

Drug fever.

Address correspondence/reprint requests to Gilles R. G. Monif, M.D., Department of Obstetrics and Gynecology, Creighton University School of Medicine, 601 North 30th Street, Suite 4700, Omaha, NE 68131, Telephone: (402) 280-4410, Fax: (402) 280-4496.

Received 19 December 1995
Accepted 18 February 1997

### DRUG FEVER

While drug fevers can be seen with a large variety of antibiotics, the incidence of drug fever is highest among patients receiving β-lactamase antibiotics.[1–4] Among the β-lactamase antibiotics, the higher incidence of drug fever occurs with the newer β-lactamase derivatives such as the piperacillin and third-generation cephalosporins.[3] It is hypothesized that the side-chains attached to core moiety are involved in the mechanism of drug fever in an additive fashion. The most common feature of antibiotic-induced drug fever to β-lactamase is a low-grade fever at the time of onset which is followed by a high and intermittent fever. The highest diurnal body temperature rises gradually and then the fever subsides promptly after the cessation of the antibiotic. This pattern of elevation is seen in 70% to 80% of all drug fevers. A transient elevation of lactic dehydrogenase can be demonstrated in approximately half the patients. While drug fevers primarily occur with β-lactamase antibiotics, the phenomenon has been described with aminoglycosides and the new generation tetracyclines.[1,2] In the majority of cases the diagnosis is inferred by the failure to find evidence of continued infectious morbidity. The diagnosis is further reinforced by a challenge experiment in which the antibiotic is discontinued and the fever disappears within the first 24 h following.

### REFERENCES

1. Feldbaum JS, Silverstein H: Streptomycin drug fever during treatment of bilateral Meniere's disease. Arch Otolaryngol 110(8):538–539, 1984.
2. Gorard DA: Late-onset drug fever associated with minocycline. Postgrad Med J 66(775):404–405, 1990.
3. Oizumi K, Onuma K, Watanabe A, Motomiya M: Clinical study of drug fever induced by parenteral administration of antibiotics. Tohoku J Exp Med 159(1):45–56, 1989.
4. Stein H, Shapiro M, Lowe J: Methicillin induced drug-fever during treatment for acute post traumatic osteomyelitis and septic arthritis. Arch Orthop Trauma Surg 102(1):23–27, 1983.
